# Case Report: Improvement in Swallowing Function Following DLPFC-Targeted Repetitive Transcranial Magnetic Stimulation in a Post-Stroke Patient with Cognitive Impairment

**DOI:** 10.3390/jcm15114055

**Published:** 2026-05-24

**Authors:** Yu Heng Yeh, Shih Heng Sun, Chang Cheng Wu, Wei Keung Lee

**Affiliations:** Taoyuan General Hospital, No. 1492, Zhongshan Rd., Taoyuan Dist., Taoyuan City 322211, Taiwan

**Keywords:** dysphagia, swallowing disfunction, post-stroke cognitive impairment, repetitive transcranial magnetic stimulation

## Abstract

**Background/Objectives:** Post-stroke dysphagia (PSD) is a common complication following cerebrovascular events, and many affected patients also present with pre-existing dementia or post-stroke cognitive impairment (PSCI). PSD primarily affects the oropharyngeal phase of swallowing, whereas cognitive impairment often compromises the oral phase. This overlap complicates diagnosis and highlights the importance of accurate assessment to guide appropriate treatment strategies. Repetitive transcranial magnetic stimulation (rTMS) has emerged as a promising therapeutic intervention for both cognitive impairment and dysphagia. **Methods:** In this study, we report a case of a patient with pre-existing dementia who experienced an ischemic stroke, resulting in PSD and PSCI. Videofluoroscopic swallowing study (VFSS) revealed oral-phase-predominant dysphagia. rTMS targeting the dorsolateral prefrontal cortex (DLPFC) was administered with the aim of enhancing cognitive function. **Results:** Following two cycles of rTMS in combination with ongoing swallowing therapy, the patient demonstrated notable improvement in both cognitive and swallowing function. **Conclusions:** Although limited by a single-case design and the possibility of spontaneous recovery, this case demonstrates that rTMS targeting the DLPFC combined with swallowing therapy may be a potential intervention for managing concurrent post-stroke dysphagia and cognitive impairment.

## 1. Introduction

Swallowing is a complex and highly coordinated physiological process controlled by an extensive neural network, including the swallowing centers in the medulla, as well as regions within the cerebral cortex, thalamus, pons, insular cortices, cingulate gyrus, and cerebellum [1]. Swallowing is typically divided into three distinct phases: the oral, pharyngeal, and esophageal phases [2]. Dysfunction in any of these phases can lead to dysphagia, which may result in complications, such as aspiration pneumonia, malnutrition, and dehydration [3]. Dysphagia also negatively impacts quality of life and is associated with increased mortality risk [4]. Dysphagia is a common complication following acute stroke, occurring in 50–80% of cases [5,6]. Although swallowing function often improves spontaneously during the early stages post-stroke, up to 13% of patients experience severe and persistent symptoms [7]. PSD is thought to result from lesions affecting both cortical and subcortical regions, particularly involving the lower motor neurons within the brainstem swallowing centers [8]. However, PSD does not always arise from direct injury to the neural substrates controlling swallowing; cognitive impairments secondary to stroke-related brain damage may also contribute to its onset and persistence. Cognitive impairment is strongly associated with oral-phase dysphagia, particularly in bolus preparation and propulsion [9,10,11]. Therefore, identifying the underlying cause of swallowing dysfunction is both challenging and crucial. Traditional rehabilitation approaches, including dietary modifications, postural adjustments, oromotor stimulation, and swallowing exercises, are commonly employed to facilitate recovery. However, outcomes are often less favorable than expected, highlighting an urgent need for new and effective therapeutic strategies for the management of PSD.

Cognitive impairment is another significant concern for patients who have experienced brain injury. Approximately 30% of ischemic stroke survivors exhibit cognitive impairment, defined as a Mini-Mental State Examination (MMSE) score of below 27 [12]. PSCI has been attributed to focal lesions in the left frontotemporal lobe, left thalamus, and right parietal lobe, which are frequently associated with vascular cognitive impairment (VCI) [13,14]. PSCI can affect multiple domains, including memory, attention, and concentration; language abilities; orientation; and executive function—with the latter playing a key role in the regulation of swallowing [15,16,17]. As a result, PSCI has been linked to unfavorable clinical outcomes, increased healthcare expenditures, greater caregiver burden, and reduced quality of life for both stroke survivors and their caregivers [18]. Rehabilitation in patients with PSCI is often more challenging, and achieving meaningful functional gains may be limited.

rTMS is a novel and increasingly utilized treatment modality in the rehabilitation of post-stroke patients. rTMS modulates cortical neuron depolarization through magnetic or electric fields [19]. It offers several advantages, including being noninvasive, well tolerated, and safe, and has shown promise in the treatment of PSCI and dysphagia [19,20]. Given the anatomical and functional overlap between cortical regions involved in cognitive and swallowing functions, some researchers have proposed that the severity of PSCI may be associated with the severity of PSD, suggesting that PSCI could serve as a predictive indicator for dysphagia outcomes. However, the role of rTMS in treating PSCI complicated by dysphagia—or dysphagia secondary to cognitive impairment—remains unclear. In this study, we applied DLPFC-targeted rTMS with the aim of enhancing cognitive function, and observed significant improvement in swallowing function. These findings suggest that rTMS may represent a promising therapeutic strategy for managing dysphagia associated with PSCI.

## 2. Case Report

### 2.1. Patient Presentation

The patient was an 86-year-old Asian woman with an educational attainment up to junior high school. She had been diagnosed with Alzheimer’s disease two years prior, with a baseline MMSE score of 23, and had been fully independent in her activities of daily living. The patient demonstrated a slow eating rate with prolonged mealtime duration. The patient used dentures and was able to chew and ingest foods of all consistencies. There were no other known underlying systemic diseases or relevant psychosocial history. The patient had no known family history of cerebrovascular disease, dementia, or cognitive impairment.

She presented with acute right-sided weakness upon waking, accompanied by drowsiness, drooling, and impaired swallowing. At triage, her vital signs were stable, but she exhibited altered consciousness, with a Glasgow Coma Scale (GCS) score of E4V3M6. Physical examination revealed right facial muscle weakness with absent gag reflex and right hemiparesis. The patient had limited cooperation during the examination, but a wet voice quality was observed. There was increased drooling with excessive salivary spillage. Oral motor examination revealed symmetrical but impaired motor function of the lips, tongue, soft palate, mandible, and dentition. The patient demonstrated delayed and infrequent voluntary swallow initiation on volitional oral motor function testing. Voluntary cough, throat clearing, and gag reflex were absent; only reflexive coughing was observed. Swallowing was effortful, with occasional choking on saliva. Neurocognitive assessment revealed severe cognitive impairment, with an MMSE score of 2. Motor assessment showed Brunnstrom stage III in the proximal and distal right upper limb, as well as the right lower limb. Laboratory testing results were within normal limits. Emergent brain magnetic resonance imaging (MRI) demonstrated acute infarctions in the left frontal and parietal watershed zones, as well as an acute infarct in the left putamen (Figure 1). Based on clinical findings, she was diagnosed with acute infarction involving the left frontal and parietal lobes and the left putamen, with resulting right hemiplegia, PSCI, and PSD. Her initial National Institutes of Health Stroke Scale (NIHSS) score was 21. Traditional rehabilitation training was initiated, and a nasogastric tube was placed to meet nutritional needs. The patient received a structured conventional rehabilitation program (30 min per session, 3 times per week) throughout the 6-month post-stroke period. The swallowing therapy focused on thermal–tactile stimulation and oral motor exercises to improve bolus transit and airway protection. Simultaneously, speech and language therapy targeted verbal communication and cognitive modulation, employing simplified task instructions to accommodate the patient’s baseline cognitive impairment.

### 2.2. Assessment Findings

After three months of standardized swallowing therapy, the patient’s GCS score improved to E4V5M6. Motor assessment revealed Brunnstrom stage V in both the proximal and distal segments of the right upper limb, as well as in the right lower limb. However, her swallowing function remained poor, with a Functional Oral Intake Scale (FOIS) score of 1, indicating no significant improvement. A videofluoroscopic swallowing study (VFSS) was subsequently performed, revealing prolonged oral transit time and severely impaired bolus formation and control across all tested volumes and consistencies. The Functional Dysphagia Scale (FDS) score was 95, indicating severe dysphagia predominantly involving the oral phase (Figure 2). FDS scoring was standardized using the first 3 mL International Dysphagia Diet Standardization Initiative (IDDSI) level 3 swallow. As no pharyngeal swallow was triggered in this case, all pharyngeal-phase parameters were assigned the maximum impairment score.

### 2.3. Intervention

Given the anticipated poor spontaneous recovery of swallowing function and the limited expectation for cognitive improvement without additional interventions, rTMS targeting bilateral DLPFC cognitive enhancement was initiated at three months post-stroke. In this case, the rTMS protocol was conducted as follows: the resting motor threshold (MT) was determined by trained clinical personnel using the relative frequency method. Electromyography (EMG) electrodes were placed over the left first dorsal interosseous (FDI) muscle. The resting MT was defined as the minimum transcranial magnetic stimulation (TMS) intensity at which at least 5 out of 10 motor evoked potentials (MEPs) exhibited peak-to-peak amplitudes of ≥50 µV. A 50% response frequency criterion was employed. The resting MT was reassessed weekly or more frequently if clinically indicated. The optimal coil position was defined as the site eliciting the maximal MEP amplitude, typically oriented at a 45° angle to the sagittal plane. The left DLPFC, corresponding to the F3 position, and the right DLPFC, corresponding to the F4 position in the 10–20 EEG system, were localized using the Beam F3 and Beam F4 methods, respectively. High-frequency (10 Hz) stimulation was administered to the DLPFC at 50% of the motor threshold, delivering 3000 pulses per session, consisting of 75 trains of 4 s duration with 26 s inter-train intervals. One treatment course consisted of 10 bilateral sessions administered over a two-week period, with one session delivered per day on weekdays. Two courses of 20 bilateral sessions each were administered in total, with a one-month interval between the courses. Treatment was delivered using a Magstim Rapid^2 stimulator equipped with a D70 alpha coil featuring active cooling. A sequential conventional rehabilitation program (30 min per session, three times per week) was administered throughout the rTMS intervention period. The patient completed two cycles of rTMS without any adverse effects within five months of the ischemic stroke episode.

### 2.4. Outcome

At approximately five months post-stroke, improvements were observed in both cognitive and swallowing functions after completion of two courses of rTMS intervention: her MMSE score increased to 9, and her FOIS score improved to 3. A follow-up VFSS demonstrated overall improvement, with an FDS score of 0 for 3 mL of IDDSI level 3 (Figure 3) and an FDS score of 0 for 1 mL of IDDSI level 4 consistency (Figure 4). Fiberoptic endoscopic evaluation of swallowing (FEES) demonstrated a Penetration–Aspiration Scale (PAS) score of 1 (Figure 5). Nine months after the stroke, her FOIS score improved to 4 and her MMSE score improved to 14. At the strong insistence of her family, the nasogastric tube was removed. Under a strict pureed diet with thickened liquids, no major adverse events were observed during the 12-month follow-up period (Figure 6, Table 1).

## 3. Discussion

Swallowing is a complex, highly coordinated physiological process regulated by an extensive neural network. This network includes the swallowing centers in the medulla, as well as regions within the cerebral cortex, thalamus, pons, insular cortex, cingulate gyrus, and cerebellum [1]. PSD is believed to result from lesions affecting both cortical and subcortical regions, particularly involving lower motor neurons within the brainstem swallowing centers [8]. Although PSD primarily affects the oral or pharyngeal phases of swallowing, it is crucial to identify the underlying etiology in each case using VFSS. In this case, the patient demonstrated oral-phase-predominant dysphagia during the early recovery period. Oral-phase dysphagia is strongly associated with cognitive impairment, likely through disruption of the voluntary cortical control mechanisms required for bolus preparation and propulsion, with deficits in frontal–executive function and memory domains showing the strongest associations with oral-phase dysfunction [9,10,11]. The oral phase of swallowing is under voluntary cortical control and requires intact cognitive functions, including attention, executive function, memory, and motor planning. Unlike the pharyngeal and esophageal phases, which are largely involuntary, the oral phase depends on coordinated cortical input from the primary motor cortex, primary somatosensory cortex, supplementary motor area, and prefrontal regions [10,21].

The prognosis of PSD is variable; however, most patients experience substantial improvement within weeks to months. Dysphagia is highly prevalent during the acute phase, affecting up to 75% of stroke patients. Although the majority regain swallowing function within the first week, approximately 11% to 50% continue to exhibit persistent dysphagia at 6 months [22,23]. Among patients with initial dysphagia, approximately 32% experience resolution within two weeks, 44% regain oral feeding prior to discharge, and only about 5% remain dependent on tube feeding at 6 months [22]. In the study by D’Netto et al., predictors of persistent dysphagia and poor recovery included penetration or aspiration identified on instrumental assessment, advanced age, bilateral lesions, lower initial FOIS scores, and greater stroke severity as measured by the NIHSS [24]. In this case, the patient presented with several risk factors for poor prognosis, including advanced age, a low initial FOIS score, and a high NIHSS score indicating severe stroke. Her rehabilitation progress appeared to plateau at approximately three months post-stroke, which may have been associated with PSCI. Given the anticipated limited spontaneous recovery of swallowing function and the restricted expectation for cognitive improvement without additional intervention, rTMS was administered to alleviate PSCI. Previous studies by Wang et al. and Chou et al. reported that rTMS was effective in treating cognitive impairment in patients with Alzheimer’s disease (AD) and mild cognitive impairment (MCI) [25,26]. Therefore, rTMS was considered a reasonable therapeutic option for PSCI, with the potential to improve swallowing function. After twelve months of follow-up, the patient’s cognitive function significantly improved, accompanied by improvements in swallowing function. This finding may indirectly suggest a possible association between her dysphagia and cognitive impairment and indicates that rTMS could be a potential intervention for patients with PSCI complicated by dysphagia. However, the results should be interpreted with caution due to possible spontaneous recovery, effect of conventional rehabilitation, and the limitations of a single-case study design.

In this study, the selection of stimulation parameters was primarily guided by safety and tolerability considerations. Patients recovering from stroke may exhibit increased sensitivity to cortical stimulation, and higher stimulation intensities or frequencies have been associated with discomfort, agitation, or reduced treatment adherence [27]. Therefore, we adopted a relatively low stimulation intensity (50% of MT) to enhance tolerability and ensure completion of the full treatment course. Despite the lower intensity, we applied high-frequency (10 Hz) stimulation over the DLPFC, as high-frequency rTMS is generally associated with increased cortical excitability and facilitation of neuroplasticity [28]. Importantly, the therapeutic effects of rTMS are not solely determined by stimulation intensity, but also by cumulative dose, number of pulses, and treatment duration [29]. In our protocol, a relatively high number of pulses per session (3000) and repeated sessions (40 sessions) were employed to achieve a sufficient cumulative neuromodulatory effect. Moreover, previous studies have demonstrated that subthreshold stimulation (<100% MT) can still induce meaningful neurophysiological and clinical effects, particularly when delivered repeatedly over multiple sessions [29]. Thus, our protocol reflects a balance between safety and efficacy, aiming to maximize clinical feasibility while maintaining therapeutic potential. In previous studies by Wang et al., the most commonly targeted regions for rTMS to improve cognitive function were the left and right DLPFC [25]. The DLPFC is a key region involved in executive functions such as working memory and cognitive flexibility. Patients with mild cognitive impairment and AD commonly exhibit executive dysfunction, which is believed to exacerbate memory deficits [26]. High-frequency rTMS applied to the DLPFC has been reported to improve cognitive outcomes [25,26,30]. In the study by Wang et al., rTMS protocols involving more than 10 sessions, multiple stimulation sites, 20 Hz frequency, and concurrent cognitive training were more effective than protocols with 10 or fewer sessions, a single stimulation site, 10 Hz or 1 Hz frequency, and no concurrent cognitive training [25]. In this case, the patient’s cognitive function showed partial improvement; however, a gap remained between her final cognitive status and baseline. It remains unclear whether her cognitive or swallowing function could have improved further if 20 Hz stimulation had been applied, or if the interval between treatment courses had been modified. In addition, spontaneous recovery and conventional rehabilitation may have confounded the interpretation of the specific effects of the rTMS intervention. In the study by Xie et al., rTMS combined with traditional rehabilitation therapy was associated with significant improvements in overall swallowing function, and combined rTMS and conventional rehabilitation may be more effective than either intervention alone for post-stroke dysphagia [31]. Thus, further studies with standardized rTMS protocols and controlled study designs are warranted to minimize the confounding effects of conventional therapy.

From a neuroanatomical perspective, this patient had lesions in the left frontal and parietal watershed regions and the left putamen. Frontal lesions are associated with impaired praxis and delayed oral transit, while parietal involvement relates to disrupted sensory integration and aspiration; putaminal damage further affects motor and sensorimotor swallowing circuits [32,33,34,35,36,37]. Similar temporoparietal dysfunction contributing to oral-phase delay has also been described in Alzheimer’s disease [38]. Functional MRI studies suggest that high-frequency rTMS over the left DLPFC enhances cortical activity and connectivity across frontal–parietal–subcortical networks, including regions implicated in this patient’s lesions, with changes correlating with cognitive improvement [39,40,41,42,43]. Taken together, these findings suggest that DLPFC-targeted rTMS may modulate a distributed frontal–parietal–subcortical network relevant to both cognition and swallowing. This supports the interpretation that the patient’s dysphagia may be related to impaired cognitive control of the oral phase. However, this relationship should be interpreted cautiously given the single-case design.

Models of PSD recovery mechanisms include the interhemispheric competition model, the undamaged hemisphere functional recombination compensation model, and the bimodal balance–recovery model [5]. However, due to differing perspectives on PSD recovery mechanisms, debate remains regarding the selection of excitatory versus inhibitory stimulation at corresponding sites [5]. Generally, rTMS for treating swallowing dysfunction involves targeting specific brain regions, most commonly the primary motor cortex (M1) [44,45,46]. The M1 houses the cortical representation of the mylohyoid, pharyngeal, and laryngeal musculature, and regulates swallowing through descending corticobulbar projections to brainstem swallowing centers, including the nucleus tractus solitarii and nucleus ambiguus [47,48]. Stimulating M1 can directly enhance the excitability of these motor pathways, promoting improved neuromuscular control and facilitating functional recovery. Furthermore, rTMS applied to M1 can modulate the interhemispheric balance between the affected and unaffected hemispheres, fostering neuroplasticity and compensatory mechanisms essential for rehabilitation [5]. High-frequency rTMS (≥3 Hz) applied to the ipsilesional hemisphere is most effective in the acute phase, while combined high-frequency ipsilesional and low-frequency contralesional stimulation or bilateral high-frequency stimulation yield the greatest improvements in the subacute phase [44,45].

The cerebellum has also emerged as an important rTMS target for PSD. It fine-tunes motor cortical and sensory inputs to produce coordinated outputs essential for the timing and sequencing of swallowing, with anatomical connections to brainstem swallowing nuclei [1]. Cerebellar rTMS increases pharyngeal motor cortex excitability bilaterally, and fMRI studies have demonstrated that it simultaneously enhances neural activity in the pons, medulla, and cerebellum [49]. Meta-analytic evidence confirms that cerebellar rTMS significantly improves swallowing outcomes, with some analyses suggesting it may be more effective than cerebral rTMS for certain swallowing measures [46,50].

In this case, the patient demonstrated a satisfactory outcome with oral-phase-predominant dysphagia following rTMS targeting the DLPFC—an area associated with cognitive function—rather than directly targeting M1 or the cerebellum. While M1 stimulation enhances corticobulbar excitability and cerebellar stimulation modulates swallowing coordination through cerebello-cortical and cerebello-brainstem pathways, DLPFC stimulation addresses the higher-order cognitive processes—including attention, motor planning, and executive function—that govern the voluntary initiation and control of the oral phase. Therefore, with careful patient selection, rTMS targeting cognitive function may improve both oral-phase-predominant dysphagia and cognitive function, particularly in patients whose dysphagia is driven by cognitive impairment rather than purely motor deficits. Nevertheless, caution is warranted when interpreting these findings because spontaneous recovery, simultaneous conventional rehabilitation, and the inherent limitations of a single-case report may have influenced the outcomes.

In this case, although the patient was unable to provide direct feedback due to cognitive impairment, family members reported subjective satisfaction with the patient’s clinical progress during rehabilitation. Specifically, improvements in cognitive function and successful NG tube removal were considered beneficial for the patient’s comfort and perceived quality of life.

## 4. Limitations

Several limitations of this study should be acknowledged. First, all parameters, including FOIS, FDS, and MMSE, were assessed by an experienced independent physician; however, allocation concealment or blinding of participants or outcome assessors was not implemented, which may have introduced an unclear risk of performance and detection bias. Second, this study was limited to the observation of improvement in a single clinical case and lacked a control group; therefore, the true benefits of this intervention remain to be determined. In addition, as this is a single-case report, the findings are inherently subject to limited generalizability, and potential confounding effects from concurrent rehabilitation or other co-interventions cannot be excluded. Furthermore, the absence of a control condition precludes definitive causal inference, and the observed improvements should be interpreted with caution, as spontaneous recovery following stroke cannot be ruled out. Third, variability in stimulation targets and the lack of standardized rTMS protocols in clinical practice may limit the generalizability and reproducibility of the findings. Therefore, future studies with larger sample sizes and randomized controlled trial designs are warranted to further evaluate the efficacy of rTMS targeting the DLPFC in improving PSCI with dysphagia and to establish standardized rTMS protocols.

## 5. Conclusions

Compared to previous studies, this case demonstrated a satisfactory outcome in a patient with oral-phase-predominant dysphagia following rTMS targeting the DLPFC—an area associated with cognitive function—rather than directly targeting M1, which is more directly involved in swallowing control. Patient evaluation, medical history taking, and VFSS remain essential for identifying the etiology of PSD and guiding subsequent rehabilitation planning. rTMS targeting the DLPFC to enhance cognitive function may serve as a potential therapeutic approach for treating PSD characterized by oral-phase-predominant dysphagia, and the observed improvements suggest a possible link between cognitive recovery and amelioration of swallowing function. However, the observed relationship should be interpreted with caution, as the findings were derived from a single-case observation without a control condition. In addition, the potential effects of conventional rehabilitation and spontaneous recovery following stroke cannot be excluded. Given the limitations of a single-case design, the absence of a control group, and variability in rTMS protocols, further studies with larger sample sizes and standardized stimulation protocols are warranted to provide more robust evidence.

## Figures and Tables

**Figure 1:**
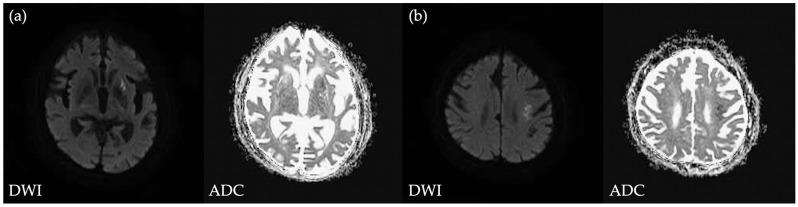
MRI demonstrated acute infarctions in the (**a**) left frontal and parietal watershed zones, as well as an acute infarct in the (**b**) left putamen.

**Figure 2:**
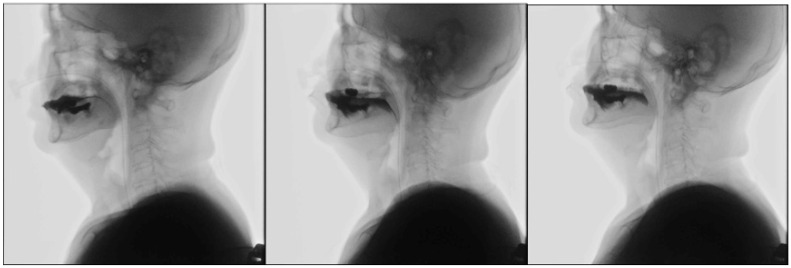
VFSS revealed prolonged oral transit time and severely impaired bolus formation and control across all tested volumes and consistencies, indicative of significant oral phase dysfunction (FDS: 95).

**Figure 3:**
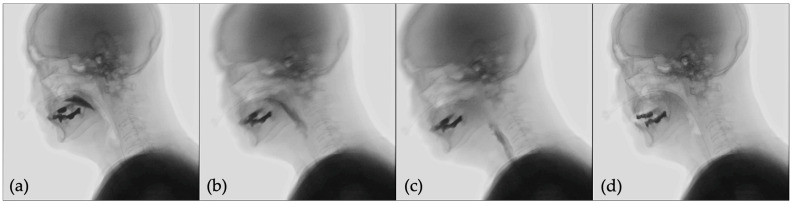
VFSS demonstrating proper bolus formation and control. For swallowing 3 mL of IDDSI level 3 consistency (FDS: 0), oral transit time was reduced to less than 1.5 s, with normal findings observed during the (**a**) oral phase, (**b**) pharyngeal phase, and (**c**) esophageal phase, and (**d**) immediately after swallowing.

**Figure 4:**
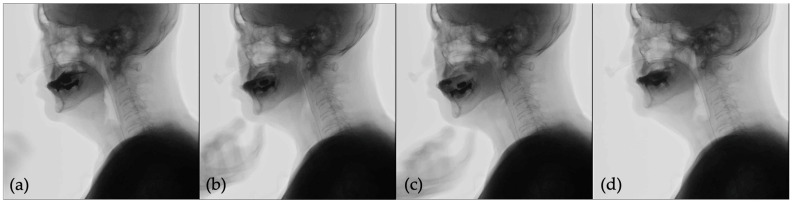
VFSS demonstrating proper bolus formation and control. For swallowing 1 mL of IDDSI level 4 consistency (FDS: 0), oral transit time was reduced to less than 1.5 s, with normal findings observed during the (**a**) oral phase, (**b**) pharyngeal phase, and (**c**) esophageal phase, and (**d**) immediately after swallowing.

**Figure 5:**
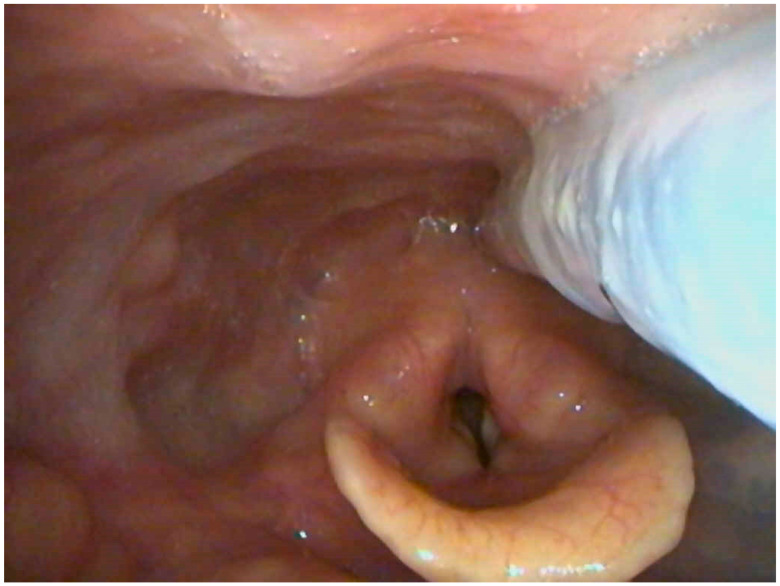
FEES demonstrated a PAS score of 1 under the first 3 mL IDDSI level 3 swallow.

**Figure 6:**
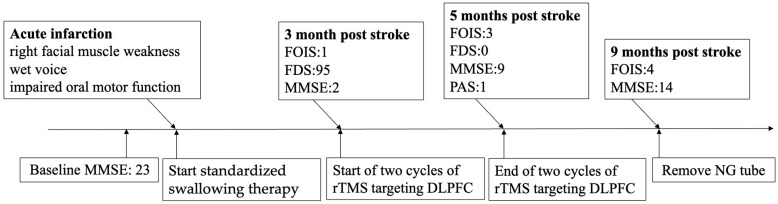
Timeline and relevant test results of the case.

**Table 1:** Comparison of Test Results Before and After rTMS Intervention.

Title 1	Before rTMS (3 Months Post-Stroke)	After rTMS (5 Months Post-Stroke)	After rTMS (9 Months Post-Stroke)
MMSE	2	9	14
FOIS	1	3	4
FDS (first 3 mL IDDSI level 3 swallow)	95	0	X
PAS	X	1	X

rTMS, repetitive Transcranial Magnetic Stimulation; MMSE, Mini-Mental State Examination; FOIS, Functional Oral Intake Scale; FDS, Functional Dysphagia Scale; PAS, Penetration–Aspiration Scale.

## Data Availability

The original contributions presented in this study are included in the article. Further inquiries can be directed to the corresponding author.
